# Supplementary material for: Effects of surface geometry on light exposure, photoacclimation and photosynthetic energy acquisition in zooxanthellate corals
Source: PLoS One. 2024 Jan 3;19(1):e0295283. doi: 10.1371/journal.pone.0295283 (PMC10763928; doi:10.1371/journal.pone.0295283)
Supplement: Attachment — Submitted filename: renamed_0f052.docx [file pone.0295283.s003.docx]

Response to the renovated manuscript

The conclusion of this paper is rather simple and important. In order to derive that, it is enough to understand only part of the results. I question some methods/calculations used here, some were used for the first time with no reference or direct measurement to assure its accuracy and some calculations and extrapolations of the data are known from previous studies but are not significant for the purpose of this study.

The authors should think carefully if using these data will strength or weakens the study, especially when the title of this study mentions photoacclimation and photosynthesis, and the derivations like PUES and Ca are not central for the final conclusion.

The title of this study should be more specific to the coral used for this study Orbicella faveolata, and not zooxanthellae corals?

This study concludes that colony geometry plays an essential role in shaping the energy balance. what is missing here is the deduction of this conclusion to the actual morphologies in nature. If these corals form vast colonies, then what percentage of the colony suffers from excessive irradiance and light-limiting conditions according to its angles?
