# Supplementary material for: Effects of surface geometry on light exposure, photoacclimation and photosynthetic energy acquisition in zooxanthellate corals
Source: PLoS One. 2024 Jan 3;19(1):e0295283. doi: 10.1371/journal.pone.0295283 (PMC10763928; doi:10.1371/journal.pone.0295283)
Supplement: Attachment — Submitted filename: Response to reviewer.docx [file pone.0295283.s004.docx]

Dear Editor,

Thank you for inviting us to undertake a second round of revisions of our manuscript. We appreciate the positive comments provided by the reviewers. Below are our point-by-point responses (in regular text) to the new comments provided by one of the reviewers (*in italics* and text of grey color). Newly added page and line numbers, which reflect where changes occur in the file with tracked changes, appear enclosed in brackets.

The reviewer wrote: *The conclusion of this paper is rather simple and important. In order to derive that, it is enough to understand only part of the results. I question some methods/calculations used here, some were used for the first time with no reference or direct measurement to assure its accuracy and some calculations and extrapolations of the data are known from previous studies but are not significant for the purpose of this study.*

*The authors should think carefully if using these data will strength or weakens the study, especially when the title of this study mentions photoacclimation and photosynthesis, and the derivations like PUES and Ca are not central for the final conclusion.*

**Authors’ reply:** We appreciate the reviewer's acknowledgment of the key conclusion in our paper. Regarding the suggestion that only a portion of the results adequately supports our conclusions, and that including the PUES analysis may weaken our study as it is not significant for the purpose of this study, we appreciate the reviewer's opinion but respectfully disagree. Our study comprises three integral components—changes in light exposure, photoacclimation, and photosynthetic energy acquisition with the inclination angle of coral surfaces, each supported by corresponding sections in the Methods and Results. It's essential to note that changes in photosynthetic energy acquisition cannot be reliably inferred solely from alterations in light exposure and photoacclimation status without considering the energy costs of repair from photodamage for the in hospite zooxanthellae. In our previously published work (López-Londoño et al., 2022), we detailed the changes in photodamage rates and the associated costs of photorepair in relation to varying light exposure levels. While we recognize that an in-depth examination of the intricate dynamics of PSII photodamage and repair processes in response to light exposure extends beyond the scope of our current study, it is important to note that the fundamental patterns have already been documented in our earlier publication. Furthermore, we are currently preparing a new manuscript that delves into a comprehensive analysis of the rates of photodamage and the related costs of photorepair in symbiotic zooxanthellae, expanding upon our previous findings and exploring these processes in greater depth.

Concerning the inquiry about certain methods/calculations used in our study to estimate the relative change in PUES, we have revised the Methods section to offer a more comprehensive description of the equations and values employed in this analysis, along with the rationale for selecting these values [lines 230-254].

The reviewer wrote: *The title of this study should be more specific to the coral used for this study Orbicella faveolata, and not zooxanthellae corals?*

**Authors’ reply:** We value the reviewer's suggestion but respectfully disagree. The light interception at a specific colony surface area is governed by geometrical relations, which exert a consistent influence on all corals and even non-living surfaces. Our primary objective in this analysis is to depict broader patterns of change in photoacclimation and energetic performance that are dictated by these geometrical relations, rather than confining the study to providing species-specific absolute values.

The reviewer wrote: *This study concludes that colony geometry plays an essential role in shaping the energy balance. what is missing here is the deduction of this conclusion to the actual morphologies in nature. If these corals form vast colonies, then what percentage of the colony suffers from excessive irradiance and light-limiting conditions according to its angles?*

**Authors’ reply:** We appreciate the insightful observation provided by the reviewer. We concur with the reviewer's acknowledgment that the percentage of the colony surface at a specific inclination angle significantly influences the energetic performance of the entire colony within a given light climate along the depth gradient. However, our study's conclusion is that "colony geometry determines light availability, holobiont physiology, and photosynthetic energy acquisition in zooxanthellate corals”. In line with this, we propose that "studying the entire coral colony and its metabolic gradients should be a research priority to enhance our understanding of colonial responses to environmental cues and the role of colony morphology and energetic economy on the vertical distribution of species and niche diversification in coral reefs." This direction not only aligns with our findings but also resonates with the comment made by the reviewer, underscoring its relevance and potential impact in the field.
